# Facilitators and barriers to healthy eating in a worksite cafeteria: a qualitative study

**DOI:** 10.1186/s12889-021-11004-3

**Published:** 2021-05-22

**Authors:** Dalia Stern, Ilian Blanco, Lucy A. Olmos, Joel J. Valdivia, Archana Shrestha, Josiemer Mattei, Donna Spiegelman

**Affiliations:** 1 CONACyT-Center for Research on Population Health, National Institute of Public Health, Cuernavaca, 7ª Cerrada Fray Pedro de Gante # 50, Col. Sección XVI Tlalpan, 14080 Mexico City, Mexico; 2 Lown Scholar, Department of Global Health and Population, Harvard T.H. Chan School of Public Health, Boston, MA USA; 3 Center for Research on Population Health, National Institute of Public Health, Cuernavaca, Morelos Mexico; 4 Department of Epidemiology, Harvard T.H. Chan School of Public Health, Boston, MA USA; 5 Department of Chronic Disease Epidemiology, Yale School of Public Health, New Haven, CT USA; 6 Department of Public Health, Kathmandu University School of Medical Sciences, Dhulikhel, Nepal; 7 Institute of Implementation Science and Health, Kathmandu, Nepal; 8 Department of Nutrition, Harvard T.H. Chan School of Public Health, Boston, MA USA; 9 Center for Methods in Implementation and Prevention Science, Yale School of Public Health, New Haven, CT USA; 10 Department of Biostatistics, Harvard T.H. Chan School of Public Health, Boston, MA USA; 11 Department of Global Health and Population, Harvard T.H. Chan School of Public Health, Boston, MA USA; 12 Department of Biostatistics, Yale School of Public Health, New Haven, CT USA

**Keywords:** Facilitators, Barriers, Healthy eating, Cafeteria, Worksite, Cardiometabolic diseases

## Abstract

**Background:**

Worksite-based nutrition interventions can serve as access points to facilitate healthy eating and translate existing knowledge of cardiometabolic disease prevention. We explored perceptions, facilitators, and barriers for healthy eating in a cafeteria at a large worksite in Mexico City.

**Methods:**

We conducted an exploratory qualitative study in a large department store in Mexico City with ~ 1500 employees. We conducted eight focus group discussions (FGD) with 63 employees stratified by job category (sales, maintenance, shipping, restaurant, cafeteria, administrative staff, and sales managers). Employees were invited to participate in the FGD if they were at the store at the day and time of the FGD for their job type. FGDs were audio-recorded, transcribed *verbatim* and analyzed using the thematic method. This process involved the researches´ familiarizing themselves with the data, generating initial codes, searching for themes, reviewing the themes, defining and naming themes, and then interpreting the data.

**Results:**

Employees defined healthy eating as eating foods that are fresh, diverse, and prepared hygienically. The most commonly reported facilitators of healthy eating at the worksite were availability of affordable healthy food options and employees’ high health awareness. Major barriers to healthy eating included unavailability of healthy foods, unpleasant taste of food, and preference for fatty foods and meat. For lower-wage workers, affordability was a major concern. Other barriers included lack of time to eat work and long working hours.

**Conclusion:**

A broad range of factors affect healthy eating at the cafeteria, some related to nutrition and some related to the employees type of job. Availability of healthy, hygienic, and tasty food at an affordable price could lead to healthier food choices in the worksite cafeteria. These strategies, along with work schedules that allow sufficient time for healthy eating, may help improve dietary behaviors and health of employees.

**Supplementary Information:**

The online version contains supplementary material available at 10.1186/s12889-021-11004-3.

## Introduction

Cardiometabolic diseases, such as cardiovascular disease (CVD) and diabetes, constitute the leading cause of global mortality and are a major contributor to reduced quality of life [1–3]. Nearly 80% of global cardiometabolic deaths occur in low- and middle-income countries where cardiometabolic burden is on the rise as a result of an epidemiological and nutrition transition [4, 5]. In Mexico, 75.2% of the population is classified with overweight or obesity [6]. Moreover, cardiometabolic diseases, are the leading causes of death and disability, where about 46% of the CVD cases and 27% of diabetes cases are attributed to unhealthy dietary habits [7]. A healthy diet is a major means of prevention of cardiometabolic diseases and mortality [8, 9]. Yet, one of the most critical issues impeding improvements in cardiovascular health, is the gap between what we know can improve health, and how it gets implemented in public health practice.

Workplaces are valuable access points for delivering interventions targeting cardiometabolic disease prevention [10]. First, worksites provide access to a relatively stable group of adults. In Mexico, 78% of adult men and 44% of adult women are employed in the formal sector [11]. Second, workers spend most of their waking hours at work [12]. In Mexico, adults spend on average 41.3 h per week at work. Third, worksite-based interventions, compared to individual-based interventions where nutrition and health primarily depend on individual choices, have the potential to be more sustainable due to social networks, the presence of worksite cafeterias providing meals for employees, and the availability of infrastructure for disseminating information [13, 14].

Worksite-based cafeteria interventions can improve eating behaviors [15] and decrease cardiometabolic disease risk [16], yet, the first step to designing an intervention is to understand the worksite food environment to identify potential intervention components, as well as the facilitators and barriers to implement those interventions. Because each worksite has its own complex environment, we conducted a qualitative study to explore perceptions about healthy eating and to understand the existing barriers and facilitators to healthy eating in the cafeteria of a large department store in Mexico City. The results of this study will inform the development of a culturally acceptable worksite-based cafeteria intervention to reduce cardiometabolic risk among Mexican workers.

## Methods

### Study design and setting

This is an exploratory qualitative study conducted from December 2018 to February 2019 in a department store in Mexico City that employs approximately 1500 employees. We based our research on the Health Belief and the Social Ecological Models. The Health Belief Model is designed to explain and predict health behaviors by focusing on the attitudes and beliefs of individuals [17]. The Social Ecological Model, on the other hand, incorporates multiple determinants into different levels of influence on behavior (i.e., intrapersonal, interpersonal, organizational, community and public policy) and considers the interaction of behaviors across these different levels of influence, which leads to multi-level suggestions for interventions to effectively change behavior [18]. We selected this store because a) the cafeteria is managed by the store and thus, the ability to improve it is enhanced, compared to out-sourced worksite cafeteria settings; b) the store employs diverse job types (e.g., from manual to desk jobs); and c) it is classified as a large enterprise based on the number of employees and stores owned by the same management across Mexico, allowing for possible scale up to other stores. Written informed consent was obtained from all participants. The study was approved by the Ethics and the Research Institutional Review Boards at the National Institute of Public Health in Mexico.

The store cafeteria cooks the meals on-site for the store employees, and it is operated by 13 cafeteria staff. The cafeteria serves about 1000 meals per day in three shifts: 1:00 pm, 2:30 pm, and 4:00 pm, and costs about $2.00 US dollars ($38.00 Mexican pesos). The cafeteria does not serve breakfast or in-between snacks. A typical menu of options includes soup (usually chicken broth with rice or vegetables), two main dishes (to pick one, usually one of them is red meat stew, and the other one a healthier chicken based), white rice, beans, white bread, corn tortillas, *agua fresca* (fruit, water, and added sugar), tea or coffee, and dessert (*pan dulce* or pastries). Sugar-sweetened beverages are available for purchase from a vending machine. Vegetable oil is used for cooking.

### Recruitment

We worked with the communication department headquarters of the store to organize the focus group discussions (FGD). To allow honest sharing of opinions and to capture the perception and experiences of everyone at the store, we decided to stratify the FGD by job type [19]. Job types were defined by the store as follow: sales, maintenance, shipping, administrative, sales managers, customer’s restaurant (not for employees), and cafeteria staff. Then, through the communication department, we collaborated with the human resources (HR) department of the store to organize the FGD. The HR department printed and placed flyers 2 weeks before the first FGD in places where they deemed adequate for all workers to see. The HR department also set the date and time for the FGD based on employees’ schedules. The HR department invited the various managers to inform their teams about the study and to invite them to participate if they were at the store at the day and time of the FGD for their job type. The order of the FGD was defined by the company. Employee’s participation was voluntary, and employees were informed that their decision to participate would not affect their employment status.

### Focus group procedures

We conducted eight FGD with 63 participants stratified by job type: 16 sales (2 FGD, 8 participants each), 12 maintenance, 5 shipping, 8 administrative, 6 sales managers, 9 restaurant, and 7 cafeteria staff. A FGD guide was developed in Spanish with input from the research team, and pilot tested for clarity (Supplementary File 1). Three main domains were explored using open-ended questions: 1) perception of healthy and unhealthy foods, 2) facilitators and barriers for healthy eating, and 3) feasibility of participating in a future intervention. We asked about absolute preferences and perceptions, and probed specific concepts when not provided by participants to elicit further comments. Questions included ‘Among the foods available in the cafeteria, which of them do you consider “healthy”? Why do you consider them healthy?’; ‘Among the foods available in the cafeteria, which of them do you consider “unhealthy”? Why do you consider them unhealthy?’; ‘What can make it possible for you to make healthier choices in the cafeteria?’; ‘What impedes you from making healthier food choices in the cafeteria?’; ‘What healthy foods can be added?’; ‘What unhealthy foods can be substituted?’

Prior to each session, participants self-reported demographic information, lifestyle behaviors, height and weight, and presence of obesity and chronic diseases on a brief questionnaire. All FGD were conducted in Spanish in a private space at the store to ensure confidentiality and facilitate honest sharing of opinions. Each session began with an introduction that included a brief explanation of the study, the FGD protocol, and ethical considerations. At the end of each session, the moderator provided a summary to confirm responses with participants. The FGD were audio-recorded and lasted for 60–90 min. The moderator (IB) and co-moderators (DS, LAO, JJV) debriefed together and took additional notes after each session. We revised the guide for the cafeteria staff FGD to include the experiences, opinions and perceptions the staff has towards their job and their interactions with cafeteria users.

### Qualitative data analysis

Audio recordings from the FGD were transcribed verbatim in Spanish by two trained native speakers (LAO, JJV) [20–22]. To ensure quality control, IB independently reviewed the transcripts against the audio recording for potential discrepancies or incomplete data. We analyzed the data using the thematic framework method [23, 24]. First, researchers familiarized themselves with the data, generating initial codes and themes. Themes were then reviewed, defined, and named for interpretation. Specifically, the analysis began by reading the FGD text several times to obtain an overall understanding of the content. Then, the team developed and Excel matrix based on the pre-defined domains and inductively coded the transcripts to identified the key emerging themes within domains, first independently and then jointly (LAO, JJV, IB), until consensus was reached. All transcripts were manually coded. Barriers were defined as factors that prevent or inhibit an individual from making a healthy decision in the workplace, while facilitators were those that promote or enable an individual to make such decisions. The analysis was conducted for all job types together, while identifying salient themes per job type. In order to illustrate the response that participants had for a specific theme, we selected and translated to English the quote that best described each relevant theme. Descriptive socio-demographic data were analyzed using Stata 14.

## Results

### Participants’ characteristics

The mean age of participants was 40 years (SD 11), 62% were female, and 27% of participants had completed an advanced technical or a bachelor’s degree. The mean body mass index (BMI) based on self-reported weight and height was 26 kg/m^2^ (SD 4), with a combined prevalence of overweight and obesity of 55%. Current smoking was reported by 18% of participants. The prevalence of self-reported medical diagnosis of hypertension, diabetes, high cholesterol, and CVD was 19, 9, 22 and 11%, respectively (Table 1). These characteristics are similar to the Mexican population.

**Table 1:** Characteristics of workers in a department store in Mexico City participating in focus group discussions about healthy eating (*n* = 63)

	n	%
**Age, years**^a^	40.0	11.1
**Female**	39	61.9
**Job type**		
Maintenance	12	19.0
Shipping staff	5	7.9
Sales staff	16	25.4
Sales manager	6	9.5
Administrative staff	8	12.7
Restaurant staff	9	14.3
Cafeteria staff	7	11.1
**Education**		
Primary (6 years)	6	9.7
Secondary (9 years)	19	30.7
High school (12 years)	20	32.3
Advanced technical	4	6.5
Bachelors	13	21.0
*Missing*	*1*	
**Household size, n**^a^	3.7	1.7
**Frequency of cafeteria use**		
Never	7	11.5
< 1 week	11	18.0
1–2 week	11	18.0
3–4 week	16	26.2
1 day	7	11.5
> 1 day	9	14.8
*Missing*	*2*	
**BMI, kg/m**^**2**a^	26.2	4.3
**BMI categories**		
Normal	26	44.8
Overweight	26	44.8
Obesity	6	10.3
*Missing*	*5*	
**Smokers**	11	17.7
*Missing*	*1*	
**Medical diagnosis of hypertension**	11	19.3
*Missing*	*6*	
**Medical diagnosis of diabetes**	5	8.8
*Missing*	*6*	
**Medical diagnosis of high cholesterol**	12	21.8
*Missing*	*8*	
**Medical diagnosis of cardiovascular disease**	6	10.9
*Missing*	*8*	

Values are counts and column percentages for categorical variables

^a^Values are means ± SD for continuous variables

### Perception of healthy eating

All emergent themes with corresponding representative quotes are presented in Table 2. Healthy eating was often described in terms of freshness, balanced, diverse, and hygienically prepared foods. Fruits and vegetables were the most commonly mentioned healthy foods. Lack of variety was recognized as a source of dissatisfaction and unhealthiness in the cafeteria. Most employees acknowledged that cafeteria meals were meat and carbohydrates and few vegetables. Lack of hygiene protocols in food preparation was associated with unhealthy foods, and some participants even reported gastrointestinal problems after eating the cafeteria food multiple times. In terms of food safety, some employees suggested that the store cafeteria should be certified to guarantee the healthiness and quality of their food, as they have for the restaurant in the store, which serves the customers, rather than the workers.

**Table 2:** Summary of resulting themes and representative quotes from cafeteria users and staff in a department store in Mexico City participating in focus group discussions about healthy eating

Pre-defined domains	Themes	Summary	Representative quotes
Perception of healthy eating	Freshness, balanced and variety	Most meals were based on meat and carbohydrates with not enough vegetables. Fruits and vegetables were the most commonly mentioned healthy foods.	“They are not balanced meals, it is either fried, or rice and pasta (Sales staff)”“We need more variety (of foods) because it is usually limited to red meat, marinades, red sauce, green sauce (Sales managers)”
	Hygiene	Lack of hygiene protocols and some people reported gastrointestinal problems.	“Sometimes they have the pots with food on the floor and the glasses and utensils are dirty (Sales manager)” “If they give us a lot of food, we need to be careful. It is not a good sign, we can get sick. We all got sick the next day after having the leftovers (Maintenance staff)”
	Nutrient and food composition	Healthy food was related to fruits and vegetables, low salt, and low fat. Foods high in oil, salt, or sugar were considered unhealthy, although traditionally Mexican and inexpensive.	“You have to use three four napkins to remove the amount of fat on the food (Sales staff)”“It would be healthier to cook with less fat and less salt, more vegetable steamed options, a little more natural (Shipping staff)”“For me the food is very garnachera (traditional street dish composed of a fried tortilla topped with various ingredients). I know that the Mexican diet is like this, tacos and all that stuff, but for me it is very heavy and fatty. It seems that they cook that a lot, maybe because it is cheap. But it is always very very fatty (Sales staff)”
	Quality	Served meals that contained hardened meat, dry bread, or burnt tortillas, as well as foods stored overnight, were deemed of poor quality.	“They serve us food from 2 or 3 days ago and it doesn’t taste good (Maintenance staff)”
Facilitators to healthy eating	Availability of healthy food^1^	Availability of healthy food options was a major motivator for healthy eating.	“I lean more towards vegetables, having more vegetable options in the cafeteria would be great (Administrative staff)” “I believe that if we get into the habit of eating vegetables, we can change (Sales staff)”
	Health awareness^2^	Employees were aware of their health, especially older participants and employees with higher occupational levels.	“The obesity problem in Mexico is because people have everything to eat, it’s on us, nobody is being told to go and eat five tortillas and a soda (Sales staff)”
	Price^1^	Cafeteria food was subsidized by the store. Food was also directly deducted from the payroll. Kitchen staff did not pay for food.	“The price of a meal is very good, if you go out you end up paying 70 pesos or more (Sales managers)”“A salad in the street has lettuce, a slice of tomato, some cheese strips, some ham strips, and it is small and costs around thirty five pesos (1.85 UDS). I think the worksite cafeteria could charge fifty pesos (2.63 UDS) if they will offer us healthier foods (Administrative staff)”
	Nutrition education^2^	Useful not only to better understand what workers should eat at work, but also to extend this knowledge to their families.	“We need to receive information if you want to have an impact, provide hard data, explain why a food is good or bad for our health (Administrative staff)” “We need to receive information on healthy eating and disease prevention if you want to have an impact on our dietary behaviors and health, provide hard data, explain why a food is good or bad for our health (Administrative staff)”
		Cafeteria staff pointed out that informing cafeteria users about the importance of healthy eating, since the information would support changes in the foods offered.	“It should be publicized: the company is concerned about your health, you will pay the same price in the cafeteria, but will have healthier food options (Cafeteria staff)”
Barriers to healthy eating	Taste^2^	Some healthy options were sometimes offered, but they were not tasty.	“We should pay more attention on how food is being cooked. Here we call it chicken or tuna salad, but it’s really lettuce and mayonnaise, it is not something people want to eat … If we serve them lettuce and chicken or tuna with mayonnaise, obviously people will not like it (Kitchen staff)” “… they serve salads but without creativity, they only put mayonnaise and lettuce (Sales staff)”
	Food preferences^2^	There is a preference for fatty foods and meat. Fatty food is part of the “Mexican culinary culture” and it is usually cheap. Cafeteria staff pointed out that when healthy foods are offered, employees did not consume them.	“When you ask workers, they will say they want vegetables, but when vegetables are offered, they complain and they ask for the meat (Cafeteria staff)” “The boss came to eat and I offered him vegetables, but he did not take them. He wanted the French fries and rice (Cafeteria staff)”
	Job intensity^1^	Eating healthy is only for certain types of workers, mainly those who have sedentary or office jobs. More physically active workers required foods that satisfy them. There was a perception that in order to perform intense work it is necessary to eat and drink a lot of carbohydrates.	“An executive spends most of his time sitting, so they know that if they eat a lot of food they may compromise their health. But here, in the kitchen, we spend most of our time standing. Sales staff are also always standing, we need more fulfilling foods, like bread and meat (Cafeteria staff)” “If I eat vegetables, I get hungry again after a while (Sales staff)”
	Food quality^1^	Offerings has too much meat and carbohydrates, and not enough vegetables; food was cooked with a lot of fat.	“It is meat daily, either beef or pork... from my perspective I do not think it is healthy to eat meat so many times a week (Cafeteria staff)”
	Price^1^	For low wage workers maintenance and shipping staff, food was not cheap. They were eating the leftovers, raising the issue of food price inequity.	“I don’t earn enough money to pay for the food in the dining room, the cost is so high (Maintenance staff)” “They are serving us leftovers … we don’t have the food made that day... the least they can do is charge us half the cost for this food... because it is not fair that we pay the same as others who are eating food made that day (Maintenance staff)”
	Time^1^	For some type of workers, lunch may be their only meal. They had no time to prepare breakfast or dinner due to long working hours and commuting.	“We usually only eat one meal a day. Once I get back home from work, I want to sleep (Restaurant staff)”
		Employees has long working hours and lack of breaks, so they had to eat more carbohydrates and fat to fill them up.	“We work 10–12 h shifts, with 1:30 h break for lunch. But after lunch and before going home we don’t get breaks (Sales staff)”
		For some type of workers, there was not sufficient time to eat within workplace; meal schedules were not respected. They could not eat at a defined time, nor take the time to eat calmly.	“I believe that a person, who is not well cared for, cannot work well, and if a person is not fed, it leads to stress with the customers. If we do not eat, we provide bad service even if we don’t want to (Restaurant staff)”
	Lack of kitchen personnel and infrastructure^1^	Cafeteria staff believed it is necessary to hire more kitchen staff to cook and serve the more than one thousand employees.	“We are few people working in the kitchen. The staff is 16, and 3 people are off every day (Cafeteria staff)”
		Cafeteria staff believed it was necessary to have better utensils and appliances.	“We have to use of oil because otherwise the food sticks on the pan (Cafeteria staff)”

Facilitators and barriers were classified based on the Socio Ecological Model as community^1^ or individual^2^

“We need more variety (of foods) because it is usually limited to red meat, marinades, red sauce, green sauce (Sales managers)”.

“If they give us a lot of food, we need to be careful. It is not a good sign, we can get sick. We all got sick the next day after having the leftovers (Maintenance staff)”.

Healthy eating was also associated with the nutrient, food composition and quality of the food, including the availability of fruits and vegetables, low salt, and low fat items. Foods high in oil, salt, or sugar were considered unhealthy, although traditionally Mexican and inexpensive. Moreover, meals that contained hardened meat, dry bread, burnt tortillas, and foods stored overnight were deemed of poor quality.

“For me the food is very *garnachera* (traditional street dish composed of a fried tortilla topped with various ingredients). I know that the Mexican diet is like this, tacos and all that stuff, but for me it is very heavy and fatty. It seems that they cook that a lot, maybe because it is cheap. But it is always very very fatty (Sales staff)”.

### Facilitators to healthy eating

Employees considered the availability of healthy food options to be a major motivator for healthy eating. Moreover, the majority of employees, especially older participants and employees with higher wages, were more aware of their health and expressed the desire to eat healthy.

“I lean more towards vegetables, having more vegetable options in the cafeteria would be great (Administrative staff)”.

One of the major themes that emerged in the discussion was the price of food. The cafeteria users, particularly those with higher wages, agreed that the price of the food in the cafeterias was reasonable, especially because food is subsidized and directly deducted from their pay checks. However, some employees questioned whether food quality could improve, given the low price. Workers with higher wages expressed that they are willing to pay more for healthier meals and better food quality.

“A salad in the street has lettuce, a slice of tomato, some cheese strips, some ham strips, and it is small and costs around thirty five pesos (1.85 UDS). I think the worksite cafeteria could charge fifty pesos (2.63 UDS) if they will offer us healthier foods (Administrative staff)”.

Another major facilitator for healthy eating reported by participants would be to receive nutrition education. Employees emphasized that the store has good communication channels (e.g., email groups, WhatsApp groups, and whiteboards) that could be used to educate employees about healthy eating. Workers stated that this education would be useful not only to help them understand and modify their eating habits, but also to extend the knowledge to their families.

“We need to receive information on healthy eating and disease prevention if you want to have an impact on our dietary behaviors and health, provide hard data, explain why a food is good or bad for our health (Administrative staff)”.

The majority of participants indicated they are willing to participate in a future cafeteria intervention.

### Barriers to healthy eating

A major barrier reported by the majority of workers was the unpleasant or bland taste of healthy food options. Some participants stated that they did not find the healthy foods tasty when they are sometimes offered in the cafeteria. For example, a salad was composed of lettuce and tuna. Thus, many participants expressed that unhealthy foods, such as fried food, tasted better than the salad. The staff cafeteria pointed out that when healthy foods were offered (e.g., salads), employees did not consume them. One of the cooks acknowledged that this was probably due to unpleasant or bland taste and lack of creativity (not visually appealing) when cooking healthy foods.

“When you ask workers, they will say they want vegetables, but when vegetables are offered, they complain and they ask for the meat (Cafeteria staff)”.

Food preferences were also identified as a barrier for healthy eating. Younger and manual workers had a preference for meat, and meals rich in carbohydrates and fatty foods. There was also a perception that healthy eating depends on physical job intensity. For example, some manual workers indicated that healthy eating is mainly for employees who have office jobs which are more sedentary, and that more physically active workers require foods that satisfy them, such as meals rich in carbohydrates and fat.

“An executive spends most of his time sitting, so they know that if they eat a lot of food they may compromise their health. But here, in the kitchen, we spend most of our time standing. Sales staff are also always standing, we need more fulfilling foods, like bread and meat (Cafeteria staff)”.

“If I eat vegetables, I get hungry again after a while (Sales staff)”.

Time was also identified as a main barrier for healthy eating for multiple reasons. First, for some workers, lunch may be their only meal because they did not have time to prepare and eat breakfast or dinner at home due to long working hours and commuting. Second, the long work shifts and the lack of breaks during working hours promoted the consumption of carbohydrates (e.g., sugar-sweetened beverages, bread) and fat-rich meals (e.g., meats) to fill them enough to last the work day. Third, the restaurant staff emphasized that their lunch time was not respected, thus, they were unable to eat at a defined time, nor take the time to eat calmly. This also raised more issues, such as not being able to treat customers well, because the staff did not feel well when they were food deprived.

“We usually only eat one meal a day. Once I get back home from work, I want to sleep (Restaurant staff)”.

“We work 10-12 hrs. shifts, with 1:30 hrs. break for lunch. But after lunch and before going home we don’t get breaks (Sales staff)”.

While the price of food was identified as a facilitator for some workers, for low-wage workers (e.g., maintenance and shipping staff), the same food was not considered cheap. Additionally, low-wage workers have earlier lunch times, thus, due to their working schedule, and because the cafeteria staff could not have the food ready for the earlier lunch time, they ended up eating leftovers, raising the issue of food price inequity.

“I don’t earn enough money to pay for the food in the dining room, the cost is so high (Maintenance staff)”.

“They are serving us leftovers … we don’t have the food made that day... the least they can do is charge us half the cost for this food... because it is not fair that we pay the same as others who are eating food made that day (Maintenance staff)”.

The kitchen staff specifically mentioned the need for additional human resources to provide healthier food options, such as salads, because it is labor-intensive to wash and cut the salad ingredients. They also commented that they need new cooking utensils to decrease the use of oil in food preparation. Finally, the kitchen staff highlighted the need to inform employees about healthy eating initiatives by the worksite and potential changes to the cafeteria food, because the success of these changes will depend on whether or not the employees accept the changes.

“It should be publicized: the company is concerned about your health, you will pay the same price in the cafeteria, but will have healthier food options (Cafeteria staff)”.

## Discussion

A broad range of factors affect healthy eating at the cafeteria, some of which are related to nutrition, and others to the job design itself, such as the amount of, and timing of, breaks. This study highlights the heterogeneity of the cafeteria experience by job type, especially for low- versus higher wages employees. Additionally, the study provides an understanding from the perspective of both employees and cafeteria staff, of what factors are perceived to facilitate dietary choices in the worksite cafeteria. Healthy eating in our study was defined by employees in terms of freshness, balance, hygiene, and nutritional quality of the foods. The participants identified foods such as fruits and vegetables as healthy and foods prepared with a lot of meat, carbohydrates and fat as unhealthy. The facilitators of healthy eating were the availability of healthy food options, workers’ health awareness, the price of food for high-wage workers, and receiving health and nutrition education. The barriers identified were individual habitual preferences for unhealthy foods, poor food quality, long working hours and job intensity, the affordability of the food to low-wage workers, and the inadequate human resources to prepare healthy foods.

Participant responses in this study are largely similar to facilitators and barriers identified in the previous literature [25]. Access to healthy food options was a commonly discussed facilitator for healthy eating across job types. Previous studies have shown that the presence of, or access to, unhealthy food options can have important adverse effects on what employees eat [26]. In contrast, access to healthy and affordable foods at work can improve employee food choices [27], thus, this is an area that can be used to drive healthy behaviors.

In this worksite, affordability was a key issue. The price of food at the cafeteria was considered to be fair for higher wage workers, and they were willing to pay more for healthy foods. However, for low-wage workers, the price of food was high, and they were not willing to pay more for healthy foods. This raises the issue of health inequalities at work [28]. A recent study provided strong evidence that subsidizing healthy foods while taxing unhealthy foods can improve food sales in the desired direction while meaningfully improving cafeteria financial outcomes [29]. While the cafeteria food at the store is already subsidized, a challenge for the worksite would be to provide access to healthy foods that are affordable to both low- and high-wage workers.

Similarity to other studies conducted in low- and middle-income countries (LMIC), we found that hygiene and food safety perceived to be important elements for healthy eating [30–32]. In fact, a facilitator identified for healthy eating in this worksite would be to have a certification, such as the he International Organization for Standardization (ISO) 9000, to ensure workers that food is prepared with the highest safety standards. Taste and food preferences were also prominently identified as factors for choosing food in the worksite cafeteria. Other LMIC studies, have also highlighted the importance of taste and food preferences for food choices in a range of settings, including worksites [30, 31, 33, 34].

According to the cafeteria staff, employees preferred and chose unhealthy foods over healthy food options, suggesting that perceptions and preferences play an important role in how likely someone is to make healthy choices [25]. This was especially true among young and workers with energy-intensive jobs, who believe they need meals rich in carbohydrates and fat to perform their work adequately and last through the day. Yet, employees who were aware of their health, such as older and higher wages workers, also highlighted the fact that the healthy foods at the cafeteria were not appealing or tasty. Thus, similar to what has been reported previously [33], efforts aimed at making healthy choices more palatable, tasty, and cooked in a more creative way might help improve healthy food choices at the cafeteria. Moreover, the worksite could establish creative communication strategies to motivate employees’ interest in healthy foods and emphasize the health benefits of choosing healthy food items. Several studies have shown that conducting group educational sessions or workshops, and environmental prompts focused on the health benefits of a healthy diet, lifestyle changes, and disease prevention, can be effective in cardiometabolic disease prevention [16, 35–37]. In fact, behavioral economics posits that patterns of behavior may result from the unconscious influences of emotional and environmental factors on individual decision-making. Thus, small changes to the environment, messaging, or intra-personal interactions can influence behavior, often through simple and cost-effective interventions such as incentives, social influence, commitment devices, and defaults. A more concerted effort to explore when and how to appropriately leverage these simple, yet effective, solutions in worksites is now warranted [38–40].

Similar to a study in the United Kingdom [41], our findings suggest that only taking a health promotion approach may not be sufficient. Some of the barriers identified in our worksite are related to organizational barriers or the job design itself, such as lack of time to eat while at work, and lack of time to eat breakfast or dinner due to long commutes and long and demanding working hours. Additionally, issues were raised regarding food inequality, where low-wage workers repeatedly mentioned that they are the ones being given the “leftover” foods. Usually, low-wage workers work the earlier shifts, making their lunchtime earlier, compared to the rest of the employees. Thus, when they go to the worksite cafeteria, the food prepared for that day is not yet ready, and they are offered the leftovers from the previous working day. This highlights some issues with the cafeteria personnel, such as not having enough time to prepare foods for the entire work force in time, and not knowing how many meals to prepare on a given day, to avoid having so many leftovers. Another barrier included the lack of human resources and appropriate cooking supplies at the cafeteria. This suggests that workplace interventions may not be sufficient if they ignore employees’ time to devote to their own wellbeing. For example, allowing more flexibility in the work day to allow employees to have time for snacks, could aid in the health and wellbeing of their workers. Yet, in order to do this, the culture and practices of the organization need to support managers to employ appropriate solutions as needed.

Our study has several strengths worth noting. To the best of our knowledge, this is the first study to explore facilitators and barriers to healthy eating in a worksite cafeteria in Mexico. Moreover, we purposely sampled workers of various job types to represent the socioeconomic diversity of workers from the same worksite. The company chosen for this study is a large enterprise in Mexico, based on the number of employees and stores across the country. Therefore, if our planned intervention is proven effective, we will be able to scale it up in other stores with a similar cafeteria setting and potentially reach a broader population [42]. Finally, involving participants in the planning stages of an intervention can help increase motivation and participation [43].

Our study had some limitations. First, we carried out this study in one store within the company; consequently, the generalizability of the findings might be limited. Secondly, we did not stratify by BMI. Previous studies have shown that overweight and obese individuals might have different eating behaviors and perceptions about healthy eating, compared to healthy weight individuals [44]. However, participants in this study had a wide BMI range, representing all body composition categories. Third, we cannot rule out selection bias. If employees who participated in this study were more interested in health issues than other workers were, the views of the sample may not represent the views of the general workforce. Finally, we used the thematic framework to analyze the data. Other frameworks, such as the grounded theory, Abductive Thematic Network Analysis, or the Deductive Critical Discourse Analysis may have given us a different result.

## Conclusion

Among employees of a large department store in Mexico City, healthy eating was defined by employees in terms of freshness, balance, hygiene, and nutritional quality of the foods. Fruits and vegetables were the most commonly mentioned healthy foods. Increasing availability of healthy food options at an affordable price and improving the quality and taste of food, combined with nutrition education, could result in healthier food choices at this worksite cafeteria. However, job intensity and lack of time will also need to be addressed by the company to help improve dietary behaviors of employees. These factors need to be considered when designing appropriate worksite-based cafeteria interventions in Mexico to promote and sustain healthy eating behaviors in order to prevent cardiometabolic diseases.

## Supplementary Information

**Additional file 1: Supplementary File 1.** Focus Group Moderator’s Guide; Description of data: Complete guide used for the focus groups

## Data Availability

The transcripts from the focus group discussion analyzed during the current study are available from the corresponding author on reasonable request.

## Abbreviations

FGD: Focus group discussions
CVD: Cardiovascular disease
BMI: Body mass index
LMIC: Low- and middle-incom countries
HR: Human resources
