# Supplementary material for: Facilitators and barriers to healthy eating in a worksite cafeteria: a qualitative study
Source: BMC Public Health. 2021 May 22;21:973. doi: 10.1186/s12889-021-11004-3 (PMC8141192; doi:10.1186/s12889-021-11004-3)
Supplement: Supplementary file 1 — Additional file 1: Supplementary File 1. Focus Group Moderator’s Guide; Description of data: Complete guide used for the focus groups [file 12889_2021_11004_MOESM1_ESM.docx]

**Focus Group Moderator’s Guide**

**date** ___________________________

**INTERVIEWER** ___________________________

**ANNotator** ___________________________

**OTHER PERSONNEL** ___________________________

**MATERIALS** Two copies of the consent form: one for the study and one for the focus group participant, refreshments, audio-recorder, pencils and notebooks for notes, computer (laptop).

**I. INTTRODUCTION (5 MIN)**

Hello. My name is [Name of interviewer.] Thank you for agreeing to participate in this conversation. This focus group is part of a research study called "Acceptability, feasibility and effectiveness of an intervention in the workplace to reduce cardiometabolic risk in Mexico." Our goal is to try to understand the feasibility and acceptability of some proposals to change the food and beverage options offered in the workplace cafeteria. This information will be used to design an intervention in the cafeteria of this company, with the objective of reducing the risk of diabetes and cardiovascular diseases of workers.

Specifically, our goal today is to hear your views on healthy and unhealthy eating, possible changes proposed to the cafeteria, and what would be the best way to implement these changes.

Recording Our discussion will be recorded because we do not want to miss any of your comments. Your name will not be included in our reports about this conversation. [Signal to the microphones.]

Confidentiality There are no correct or incorrect responses in today’s discussion. It is very important that you give us your honest opinion. During the conversation, I encourage you to share your opinions as every one of your comments is valuable. At the same time, you do not have to respond to all of the questions, and you are free to not respond to questions that you do not feel comfortable answering. Everything you say is completely confidential, and we will not report your name or any other identifying information.

I want you to feel comfortable during this discussion. You can say as much or as little as you would like for each question.

Orientation Feel free to get up and go to the bathroom which is located _____________.

I am going to ask that you please turn off your cell phone during this conversation. If you are waiting for a call, please put your cell phone on vibrate so as not to interrupt the conversation. You may leave briefly to take the call but please return as quickly as possible. Your attention to the discussion is very important for it to run smoothly.

At times, I may need to interrupt or ask for clarification. Please feel free to ask me to explain any one of my questions or comments if they are not clear.

Introductions Now we will turn on the audio-recording to begin the conversation. [Start recording]. We will start by introducing ourselves. Please tell us your first name and your favorite food (ice-breaker). Why don’t I start? [Moderator introduction.]

**II. PERCEPTION OF HEALTHY AND LESS HEALTHY FOOD AND BEVERAGES (20 MIN)**

Of the foods and beverages available in the cafeteria, which of them do you consider to be "healthy"?

*Probe 1: What makes these foods and beverages healthy?*

Of the foods and beverages available in the cafeteria, which of them do you consider to be "less healthy"?

*Probe 1: What makes these foods and beverages less healthy?*

In your opinion, is there any relationship between food and obesity, diabetes, and cardiovascular disease?

*Probe 1: Are there foods that contribute to obesity, diabetes, and cardiovascular disease?*

*Probe 2: Are there foods that help prevent obesity, diabetes, and cardiovascular disease?*

**III. FEASIBILITY AND ACCEPTABILITY TO CHANGE (20 MIN)**

In your opinion, what do you think of the foods and beverages currently offered in the cafeteria?

In your opinion, what aspects of the cafeteria help you or allow you to eat healthy?

In your opinion, what aspects of the cafeteria make it difficult or impossible for you to eat healthy?

What would motivate or help you eat healthier in the cafeteria at work?

*Probe 1: availability of food, price, taste, health*

**IV. POSSIBLE INTERVENTIONS - MOTIVATION / FACILITATORS AND BARRIERS (30 min)**

**Food and beverages offered**

What healthy foods and beverages would you like to see offered in the cafeteria?

What less healthy foods and beverages would you like to see reduced or replaced or eliminated from the cafeteria?

What do you think of the use of oil in the preparation of food in the cafeteria?

What do you think about the use of salt in the preparation of food in the cafeteria?

**Prices**

What do you think about the price of the food and beverages offered in the cafeteria?

*Probe 1: What foods and beverages are expensive?*

*Probe 2: What foods and beverages are cheap?*

If prices fell, what foods would you consume the most?

If prices increased, what foods would you consume less?

Tell me how you would react if the cafeteria increased the price of less healthy foods and at the same time decreased the price of healthy foods.

*Probe 1: What increase in price would they tolerate?*

*Probe 2: What decrease in price would appreciate?*

**Labelling**

What is a "calorie"? What is the health impact of calories?

*(Remind you that there are no right or wrong answers, we just want to know your opinion.)*

Tell me what you would think if you saw the number of calories for a specific food on the cafeteria menu.

*Probe 1 How would it affect your decision about whether or not you should eat that food or beverage?*

Tell me what you would think if you saw the type and time of exercise that you must do to burn the calories consumed in less healthy foods.

*Probe 1 How would it affect your decision about whether or not you should eat that food or drink?*

Tell me what you would think if less healthy foods were labeled red, healthy foods were labeled green, and foods that are neither healthy nor unhealthy were labeled yellow.

*Probe 1 How would it affect your decision about whether or not you should eat that food or beverage?*

**V. FEASIBILITY OF PARTICIPATION IN THE INTERVENTION (10 min)**

If we carried out an intervention in the company cafeteria to promote the consumption of healthy food and beverages, would you be willing to participate?

What would motivate you to agree to participate in the intervention?

*Probe 1: time, monetary incentives, work incentives*

**VI. SUMMARY (5 min)** Allow me to summarize briefly what we have discussed today. I heard… [Summarize, pause]

Have I summarized well what was said today? Do you have anything else to add?

**ViI. CONCLUSION** Thank you for your participation in today’s conversation.
